# *Haemophilus influenzae* porine *omp*P2 gene transfer mediated by graphene oxide nanoparticles with effects on transformation process and virulence bacterial capacity

**DOI:** 10.1186/1477-3155-12-14

**Published:** 2014-04-16

**Authors:** Julia Nogueira Varela, Maria Cecília Krähenbühl Amstalden, Rafaella Fabiana Carneiro Pereira, Luciana Maria de Hollanda, Helder José Ceragioli, Vitor Baranauskas, Marcelo Lancellotti

**Affiliations:** 1 LABIOTEC - Biotechnology Laboratory, Department of Biochemistry, Institute of Biology CP6109, University of Campinas - UNICAMP 13083-970, Campinas, São Paulo, Brazil; 2 NanoEng - NanoEngineering and Diamond Laboratory, School of Electrical and Computer Engineering, Department of Semiconductors, Instruments and Photonics, University of Campinas, UNICAMP, Av. Albert Einstein N.400, CEP 13 083-852 Campinas, São Paulo, Brazil

**Keywords:** *Haemophilus influenzae*, Graphene oxide, Bacterial transformation, Outer membrane protein, Virulence, Porin

## Abstract

**Background:**

*H. influenzae* is a natural competent bacterium that can uptake DNA from the environment and recombine into bacterial genome. The outbreaks of Brazilian purpuric fever, heavily polluted areas of a different *H. influenzae* biogroup - *aegyptius* - as well as gene transference between *Neisseria meningitis* make the transformation process an important evolutionary factor. This work studied the horizontal transference of the *omp*P2 gene from a multiresistant strain of *H. influenzae* 07 (NTHi), under the influence of graphene oxide nanoparticles in order to mimic an atmosphere rich in suspended particles and this way verify if the CFU transformants number was increased.

**Material and methods:**

In this article the gene ompP2 was transformed into different strains of *H. influenzae* mediated or not by graphene oxide nanoparticles in suspension, followed by the adhesion tests in Hec-1B (human endometrium adenocarcinoma) and A549 (pulmonary epithelial carcinoma) cells lines. The transformation frequency and the adhesion capacity were determined in all the mutants to which the ompP2 gene was transferred and compared to their wild type strains.

**Results:**

The nanoparticles increased the transformation ratio of one particular strain isolated from a pneumonia case. The adhesion patterns to A549 and Hec1b cell lines of these mutated bacteria has their capacity increased when compared to the wild type.

**Conclusions:**

Graphene oxide nanoparticles aid the transformation process, helping to increase the number of CFUs, and the mutants generated with the *omp*P2 gene from a *H. influenzae* resistant strain not only present a chloramphenicol resistance but also have an increased adherence patterns in A549 and Hec1B cell lines.

## Background

*Haemophilus influenzae* is a gram-negative pleomorphic cocobacillus that usually colonizes the upper respiratory tract in humans. This specie can be classified by two methods: biotypes and serotypes. The division into biotypes is based on the presence and production of enzymes such as ornithine descarboxylase, urease and those that act on fermentation of D-xylose and on the production of indol. With this classification, *Haemophilus influenzae* can be divided into 9 biotypes: I to VIII and *aegyptius*[1,2]. *H. influenzae* can also be classified based on the production of a polyssacharide capsule composed by a 3-β-ribose-(1-1)-ribitol-phosphate polymer (RPR), whose antigenic structure divides the capsulated strains into 6 immunologic known serotypes: a – f [3] and non-typed strains, which do not produce capsule [4]. Other methods such as membrane protein analysis, lipopolysaccharide profile and isoenzymes electrophoresis can be used for the purpose of studying and understanding the epidemiology of *H. influenzae*[5].

*H. influenzae* type b is the most invasive of all serotypes and known as the main causer of meningitis, as well as the most relevant pathogen of the upper respiratory tract in children and in adults [6-9]. *H. influenzae* is responsible for 30 to 50% of all bacterial meningitis over the world and the second most common agent of pneumonia in children [10]. The non-typed forms of *H. influenzae* (NTHi) are generally associated to moderate diseases of the upper respiratory tract in children and pneumonia in adults with Chronic Obstructive Pulmonary Disease (COPD) or Cystic Fibrosis [11]. The polyssaccharidic capsule is considered the main antigen of this microorganism [3]. Another important pathogen factor is the lipopolyssacharides (LPS) associated with outer membrane proteins (OMP), which causes high fever and coagulation disorders [12].

*H. influenzae* resistance to antibiotics increased significantly over the last 20 years. This resistance is worse to beta-lactamic drugs, due to their production of beta-lactamase [13]. Since the first reports of *H. influenzae* resistant strains to ampicillin in 1974 in the USA, the main proposed mechanism was the production of beta-lactamases TEM-1 and ROB-1 by plasmids. The prevalence of beta-lactamase producing strains increased 15, 2% from 1983 to 1984 and 31, 3% from 1997 to 1998. *H. influenzae* is not only resistant to ampicillin; they also present non-susceptibility to cephalosporin, trimethoprim, cephalosporin, tetracycline and sulfonamides [14-17].

**Figure 1:**
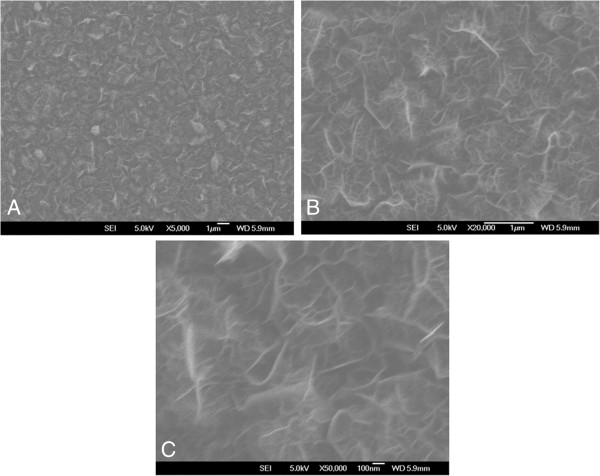
Field emission electron microscopy show in different size and magnification of graphene oxide (in A: panoramic view, B: micrometric scale and C: nanometric scale).

One of the main reasons for the emergence of multiresistant strains over the last years is the horizontal gene transfer. *H. influenzae* is considered to be natural competent, i.e., a bacterium capable of taking up exogenous DNA and incorporating it into its own genetic material by transformation [18,19]. These new genes incorporated onto the genetic code may provide mechanisms to avoid or inhibit the action of antimicrobial drugs. Even small amino acid changes in proteins can enhance the resistance of bacteria to drugs. One example is the Outer Membrane Proteins (OMP) also known as porines, which in *H. influenzae* are classified from P1 to P6, in order of their decreasing molecular weight (Figure 1) [20]. Small changes on the amino acid sequence of one of these proteins were related to an increase of antibiotics resistance in *Haemophilus influenzae* strains isolated from cystic fibrosis patients [21]. Porines isolated from resistant strains present a lower electric conductibility than wild-type porines. This would explain the non-susceptibility to beta-lactamic antibiotics and other drugs with low molecular weight [22].

The use of organic and inorganic nanomaterials is nowadays a new promising tool to biomedical researches, as for example in cancer treatment and diagnostics, drug delivery systems and bacterial processes [23]. Researches have shown that transformation frequencies and transformation process can be influenced by nanoparticles, especially mesoporous silica and carbon nanotubes [24-26]. Graphene oxide nanoparticles (GON), a layered structure of oxidized graphene sheets with epoxy- and hydroxyl groups at their edges, is a new material that attracts attention due to their distinct electronic, thermal and mechanical properties [27,28]. However, their potential role on bacterial transformation is still unknown. As graphene is composed by carbon atoms, it shows similar chemical characteristics to nanoparticles present in the atmosphere of highly polluted regions, provoking respiratory disorders when these particles are inhaled.

This work aimed to see the effect of grapheme oxide nanoparticles on the transformation frequency of *H. influenzae*; especially on the horizontal transfer of *omp*P2 amplicon obtained from NTHi serotype 07, resistant to chloramphenicol and tetracycline; as well as to verify the adhesion of mutant and wild-type strains on cells A549 and Hec-1-B in the presence of antibiotics.

## Methods

### Bacterial strains

All strains used in this work are listed in Table 1. *H. influenzae* strains were grown in chocolate agar or BHI agar (Difco) supplemented with NAD (Sigma, Saint Louis, MO, USA) 0,2 mg/ml and hemin (Sigma, Saint Louis, MO, USA) 2 mg/ml at 37°C with 5% CO_2_ for 18–24 hours.

**Table 1:** Bacterial strains used in this work

**Strains**	**Characteristics**	**Origin**
**Rd**	*Haemophilus influenzae* standard strain completely sequenced, from sorotype d	INCQS – FIOCRUZ
**Hib-βlac**	*Haemophilus influenzae* serotype b ATCC	INCQS – FIOCRUZ
**Hi07**	*Haemophilus influenzae* NTHi - CmR, TcR, ApR	LABIOTEC
**Hi38**	*Haemophilus influenzae* serotype a isolated from a pneumonia case	LABIOTEC
**Hi13**	*Haemophilus influenzae NTHi hmw*+	LABIOTEC
**Hi45**	*Haemophilus influenzae* serotype b isolated from an hemoculture	LABIOTEC
**Hi46**	*Haemophilus influenzae* serotype b isolated from a hemoculture	LABIOTEC
**Hi47**	*Haemophilus influenzae* NTHi isolated from a hemoculture	LABIOTEC
**Hic**	*Haemophilus influenzae* serotype c ATCC 9007 NCTC 8469.	IAL – SP
**Hie**	*Haemophilus influenzae* serotype e NCTC 10479.	IAL – SP
**Hif**	*Haemophilus influenzae* serotype f NCTC 7918.	IAL – SP

IAL – Adolfo Lutz Institute SP, INCQS – FIOCRUZ – National Institute of Quality Contol - Oswaldo Cruz Foundation.

LABIOTEC – Biotechnology Laboratory – IB – UNICAMP.

### Cell cultures

Cell lines A549 (pulmonary epithelial carcinoma) and Hec-1-B (human endometrium adenocarcinoma) were obtained at the Institute Adolfo Lutz, Brazil. They were cultivated at RPMI medium 1640 (Cultilab, Campinas, Brazil) with 10-20% Bovine Fetal Serum (BFS), depending the cellular lineage, and maintained at 37°C with 5% CO_2_. After the semiconfluent cell layer formation, the cells were trypsinized and transferred to a 24-well polystyrene plate. Each well received 1 ml of cell culture and the cell concentration after its trypsinization was 1.106 cells/ml. Cells were incubated under same conditions as previously described.

### Graphene oxide nanoparticles synthesis

**Figure 2:**
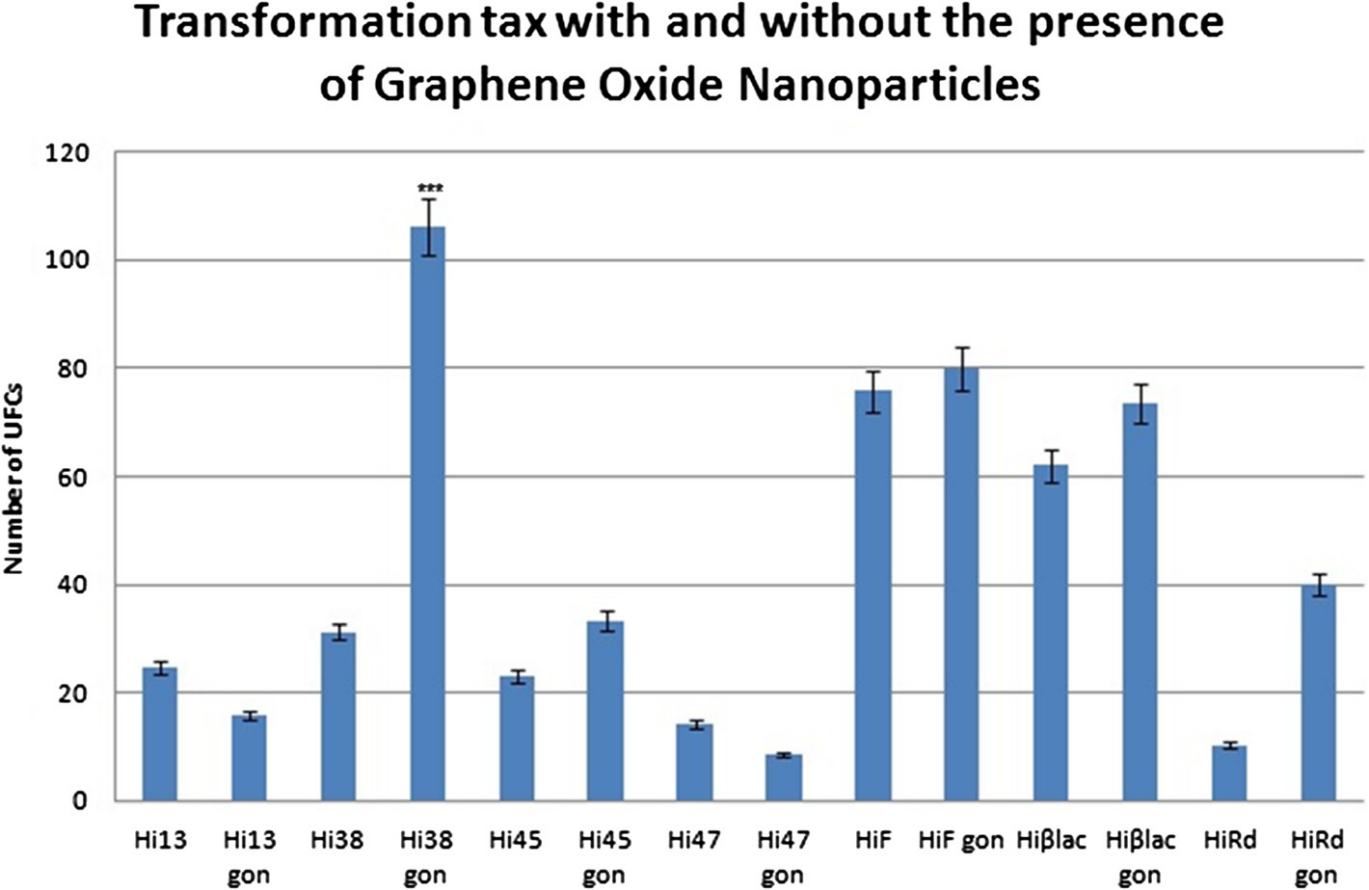
Transformation frequency with and without the presence of Graphene Oxide Nanoparticles, with p < 0,001 (***).

Graphene oxide nanoparticles were prepared after a catalytic conversion using a cupper substratum. 1 ml of a polyaniline diluted in dimethylformamide was added on the substratum and dried in room temperature for two hours. On the substratum with polyaniline was put 0,2 ml of a solution of nickel nitrate diluted in acetone. Cupper substratum was immersed into a chemical vapor deposition reactor assisted by a hot filament. The reactor contained a carbon source formed by camphor, acetone and citric acid. Raman spectrometry and Field Emission Scanning Electron Microscopy can be seen in Figure 2.

### ompP2 gene amplification

Genomic DNA from *H. influenzae* strain NTHi 07 was extracted using the protocol described by Sambrook et al. [29]. The sequence of the gene *omp*P2 was obtained at Genbank GI:2981106 (http://www.ncbi.nlm.nih.gov/nucleotide). Primers used to gene amplification are listed in Table 2.

**Table 2:** Primers used

**Primer**	**Nucleotids sequence 5**′- **3**′	**Description**
PorHi Fw	ATTAATCGTTGGTGCATTCG	*porHi forward*
Por Hi Rev	GAAGTAAACGCGTAAACCTACAC	*porHi reverse*

### Transformation process

Grown bacteria were transferred to BHI broth supplemented with NAD 2 μg/ml and hemin 10 μg/ml at a final concentration of D_600_ 1,0. Transformation process was made using 100 μl of bacterial solution incubated with 5 μl of *omp*P2 amplicon at a concentration of 1 μg/ml, followed by the addition of 15 μl of graphene oxide nanoparticles at a concentration of 20 μg/ml for 30 minutes. Afterwards, 1 ml of supplemented BHI broth was added and the transformation was incubated at 37°C with 5% CO_2_ for 3 hours. An aliquot of 100 μl was then transferred to BHI agar plate containing 2 μg/ml of chloramphenicol and incubated for 18 hours under the same described conditions. Negative control was made without the use of nanoparticles. Transformed CFU were count and statistical analysis was made by comparing the number of transformants obtained with and without the use of nanoparticles. To verify the successful gene acquisition, a PCR was made under the same conditions as previously described.

### Cytotoxicity assay

Cytotoxicity tests were made to analyze whether the antibiotic present in bacterial medium would interfere on cellular growth. After 24 hours of incubation, the medium was removed, the wells were washed 3 × with 0.1 mL of PBS Buffer (137 mM NaCl, 10 mM phosphate, KCl 2.7 mM, and a pH of 7.4). 0.2 mL of RPMI1640 was added in each well. An initial concentration of chloramphenicol of 4 μg/ml was added into the first wells and diluted into half concentration in each well. After incubation for 3 h at 37°C, the medium with dye was removed and carefully 0.2 mL of ethanol was added to solubilize the blue formazan (yielded from MTT reduction by viable cells). The plates were shaken for 10 minutes and the absorbance for each well was read in a spectrophotometer ELx800 Absorbance Microplate Reader (BioTek, USA) at λ = 570 nm. The values were expressed as percentages of MTT reduction compared to the control, where cells were not exposed to test agents [30].

### Neutral red (NR) uptake assay

The Neutral Red uptake assay was performed as described [31]. After incubation, the medium containing the dye was removed and the wells were rapidly washed with 0.2 ml of calcium-formaldehyde solution (10 ml of 40% formaldehyde, 10 ml of CaCl_2_; 10 ml deionized water). The solution was discarded and 0.2 mL of ethanol-acetic acid was added (1 ml of glacial acetic acid, 100 ml of ethanol 50%). The plate was kept for 15 minutes at room temperature and the absorbance for each well was read in a spectrophotometer ELx800 Absorbance Microplate Reader (BioTek, USA) at λ = 540 nm [32]. The values were expressed as percentages of NR uptake compared to the control, in which the cells were not exposed to test agents.

### Adhesion assay

Adhesion assay on cells was performed based on the description of Scaletsky et al. [33]. 50 μl of bacterial suspension with a concentration of 1.106 CFU/ml was added into each well containing 1 ml of RPMI 1640 with 10% BFS and 2 μg/mL of chloramphenicol. After an incubation period of 3 hours at 37°C with 5% CO_2,_ cellular medium was removed and each well was washed five times with RPMI 1640, to remove bacteria that did not adhere to the cells. 1 ml of RPMI was added to each well and its bottom was scraped with a micropipette tip to release adhered bacteria. An aliquot of 50 μl was taken and plated into chocolate agar medium with chloramphenicol for the mutant strain and without the antibiotic for wild-type strains. The plates were incubated for 18 hours at 37°C with 5% CO_2_ and CFU were counted.

### Statistical analysis

Statistical analysis was performed using GraphPad Instat program: transformation efficiency was analyzed by Bonferroni test with the number of CFU transformants with and without the presence of graphene oxide nanoparticles. Considered values were p < 0.05. The adhesion assay was analyzed by Tukey’s test with a comparison of a control sample. All experiments were made in triplicate.

## Results and discussion

### Transformation process and resulting frequencies

**Figure 3:**
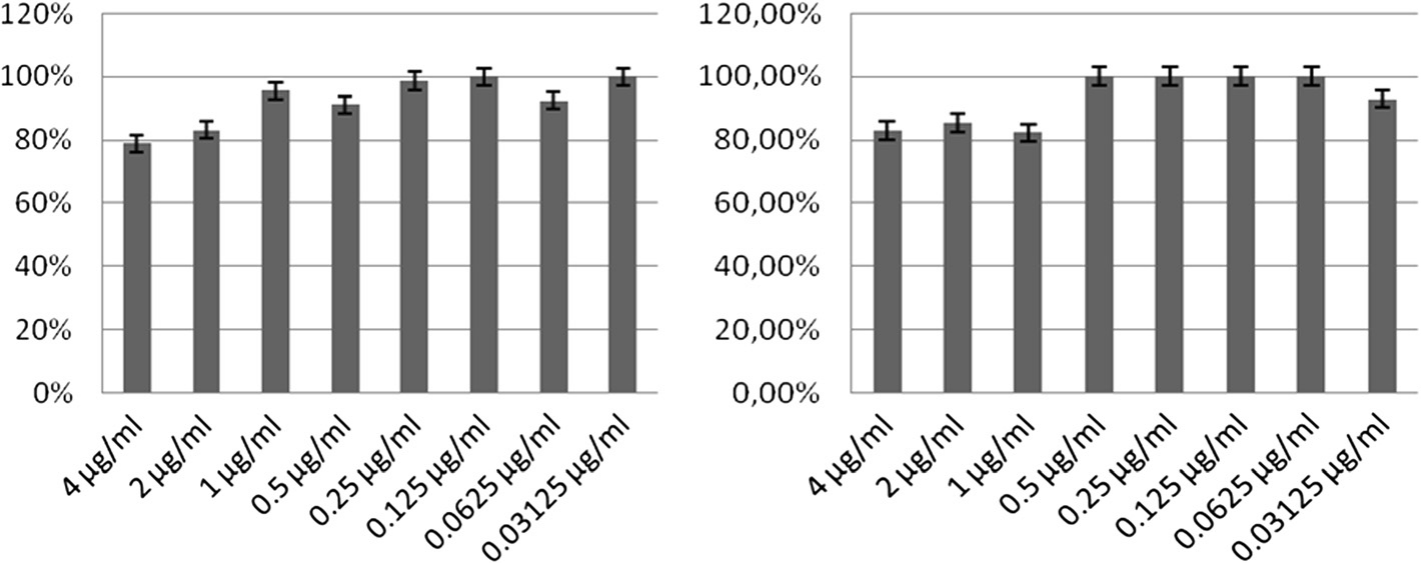
Citotoxicity in A549 and Hec1B cell lines.

The graphic of transformation frequencies with and without the use of nanoparticles for each *H. influenzae* strain is shown in Figure 3. As we can see, the transformation frequencies vary among the strains. *Hi*38, *Hi*45, *HiF*, *Hiβlac* and *Hi*Rd presented an increased transformation frequency with the presence of GON, whereas for the strains *Hi*13 and *Hi*47 these values were decreased. However, the only strain that presented an extremely significant value of p < 0.001 is *H. influenzae* 38, as shown in Table 3.

**Table 3:** Transformation tax value per colony forming unit and standard deviation

**Strains**	**CFU**	**STD**
**Hi13**	24.667	3.786
**Hi13 gon**	16	5
**Hi38**	31.333	7.234
**Hi38 gon**	106	14.177
**Hi45**	23	7.937
**Hi45 gon**	33.33	3.215
**Hi47**	14.33	1.528
**Hi47 gon**	8.667	3.215
**HiF**	75.667	16.258
**HiF gon**	80	12.49
**Hiβlac**	62	18.52
**Hiβlac gon**	73.33	22.301
**HiRd**	10.33	4.041
**HiRd gon**	40	26.889

The verification of *omp*P2 incorporation into the genomic DNA of the receptor strains was made by PCR analysis (data not shown). Studies of how nanoparticles effect transformation processes have already been made, proving to have an increase on its frequency. One hypothetical mechanism for this phenomenon could be explained by a complexation of the nanomaterial with DNA, avoiding its degradation by DNAse enzymes. The uptake of DNA and its incorporation into bacterial genetic material could be facilitated by these nanoparticles [24,25].

GON are found in high concentration in polluted atmospheres, especially in arid regions. This way, the study of their influence on *H. influenzae* transformation mimics a natural environment of carbon nanoparticles in the atmosphere, helping the transformation process in bacteria that asymptomatically colonizes the upper respiratory tract in humans. In certain regions were primitive agriculture is practiced, performed by sugar cane burning, the resulting emission of micro and nanoparticles generate a major series of respiratory complications and infections [34]. This way, the only strain which presented a statistically significant increase of transformation frequency is *H. influenzae* 38. Interestingly, this strain distinguishes itself from the other studied strains by being clinically isolated from a pneumonia sample (Table 1); because of that, this bacterium can be more adapted to a carbon nanoparticle-concentrated atmosphere and therefore m use them in its own favor than strains isolated from blood cultures. As graphene oxide nanoparticles present a similar structure and physical-chemical characteristics than multi-walled carbon nanotubes and mesoporous silica nanoparticles, it is believed that these nanostructures share the same action mechanisms in transformation processes. Therefore, the same hypothesis previously presented can be applied to explain the major increase of transformation frequency of *H. influenzae* strain 38 with the presence of graphene oxide nanoparticles.

### Cytotoxicity

**Figure 4:**
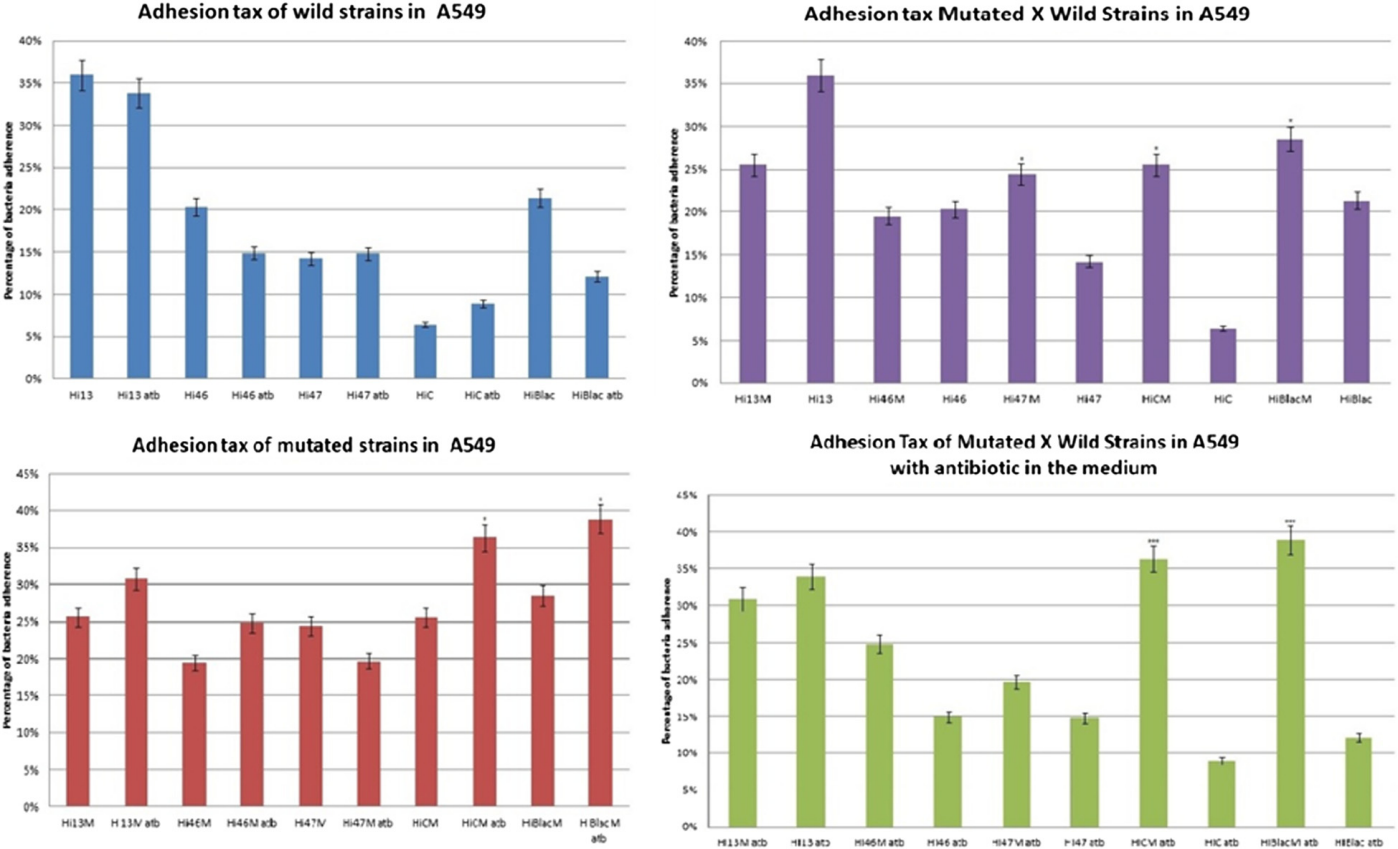
**Adhesion patterns of wild and mutated strains of *H. influenzae *in A549 cell line.** With atb- antibiotic, M- mutated, p < 0,05(*), p < 0,005 (**) and p < 0,001(***).

Graphics of cellular viability under the effects of chloramphenicol results are shown in Figure 4. The highest concentration (4 μg/ml) led to a decrease of 20% for Hec-1-b and A549. For the concentration of 2 μg/ml used in the adhesion assay, the cell viability resulted in 83%, thus chloramphenicol is not considered toxic under these conditions.

### Adhesion assay

**Figure 5:**
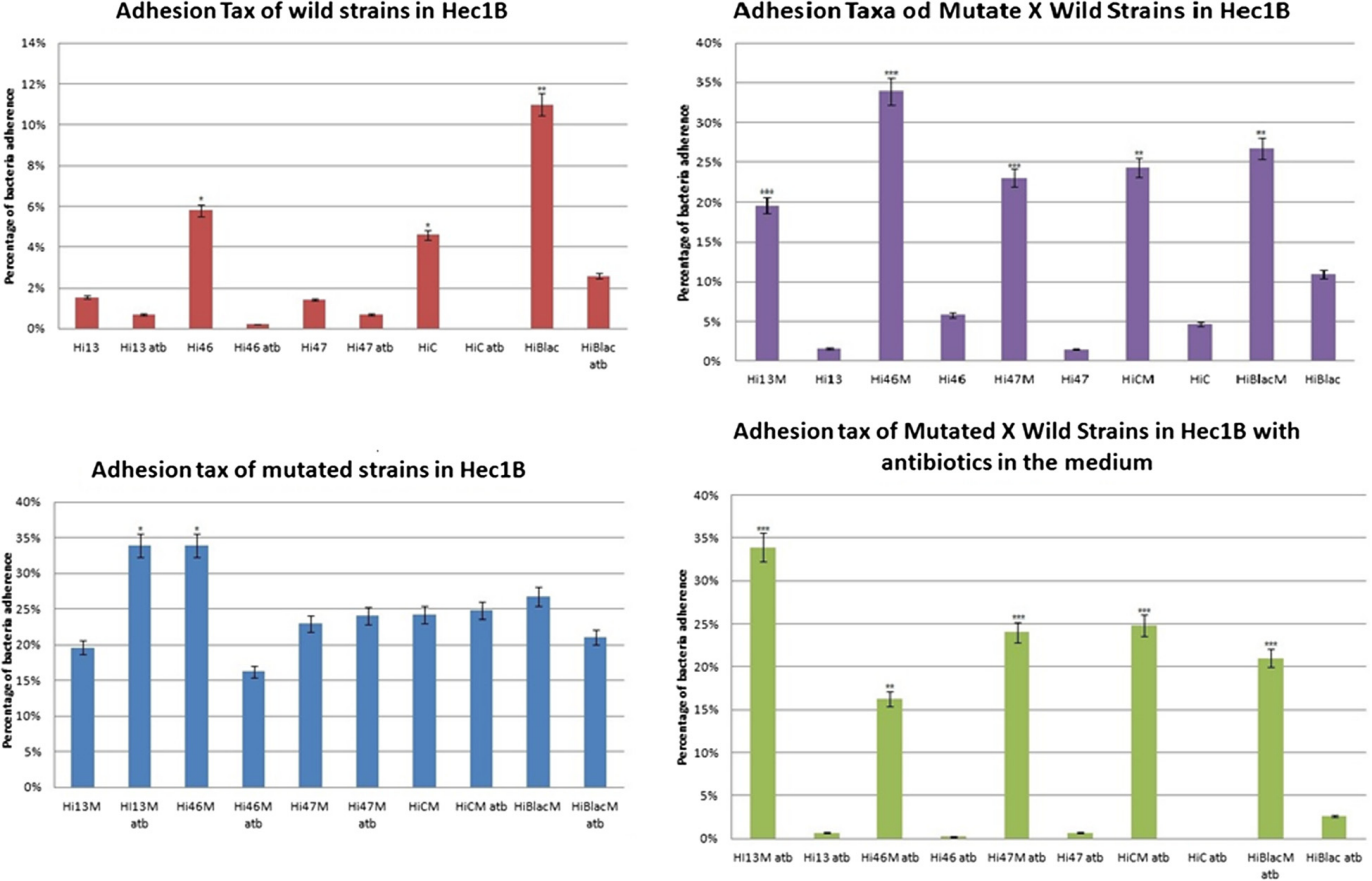
**Adhesion patterns of mutated and wild strains of *H. influenzae *in Hec1B cell line.** Atb- antibiotic, M- mutated, p < 0,05(*), p < 0,005 (**) and p < 0,001(***).

The graphics of cellular adhesion for wild-type and mutant strains in A549 and Hec1B cells with and without the presence of chloramphenicol can be seen in Figure 5. Wild-type strains of *H. influenzae* did not present any statistical relevant values for adhesion in A549 cells with or without the presence of antibiotics, while mutant strains C and βlac show an increase of adhesion when chloramphenicol was added, resulting into a significant ρ value of p < 0.05. By comparing wild-type and mutant strains, the adhesion of the first ones with and without chloramphenicol in A549 cells had an increase of p < 0.05 and p < 0.001, respectively.

As for the adhesion results in Hec1B cells, mutant strain Hi13M showed an increase of its adhesion in the presence of antibiotics when compared to the adhesion with its absence, with p < 0.05. In an environment without chloramphenicol, Hi46M also had an increase with a significant value of p < 0.05 compared to its wild-type strain. The comparison between wild-type and mutant strains with antibiotics presence revealed a clear increase of adhesion by the mutant strains. *H. influenzae* 13, 47, C and βlac mutant and wild-type strains presented an extremely significant p value of < 0.001, while 46 strain had a very significant value of p < 0.01.

The adhesion assay to see the influence of mutation of *omp*P2 gene was proposed due to the importance of bacterial adherence at the mucosal surface on host organisms as a virulence factor [33]. The pulmonary adenocarcinoma cell lineage A549 was chosen due to *H. influenzae* predilection of respiratory tract [31], while Hec1B cells of endometrial adenocarcinoma were used with the purpose of mimetizing an endothelium [35]. As cytotoxicity results presented a cellular viability of over 80% at the highest concentration used of chloramphenicol (2 μg/ml), it would not affect the cellular growth of neither A549 nor Hec1B lineages. *omp*P2 provides *H. influenzae* with resistance to chloramphenicol, thus the presence of this antibiotic in the medium increases the gene expression in mutant strains, at the same time it inhibits the bacterial growth in wild-type strains. Therefore, it was already expected that mutant strains presented a higher adhesion in cell cultures than wild-type ones. Nonetheless, the adhesion taxes without the presence of antibiotics also presented increased results for the majority of mutant strains, with a significant value of p < 0.05 for Hi47M, HiCM and HiβlacM in A549 cells, while all mutant variants showed highly significant ρ values of p < 0.01 and p < 0.001 in Hec1B cells.

Bacterial strains presented different adhesion taxes on A549 and Hec1B cells. While all mutant strains showed a major adhesion with and without antibiotics in Hec1B cells, with a significant ρ value when compared to their wild-type, this behavior could not be seen in A549 cells. One possible explanation is the fact that *H. influenzae* is a commensal bacterium of the upper respiratory tract and therefore wild-type strains present a natural capability of adhesion on these cells. On the other hand, these bacteria only have contact with endothelial cells in meningitis cases. As this microenvironment does not permit a direct transition from one host to another, the adhesion on Hec1B does not present any natural evolutionary potential for *H. influenzae*[36,37]. The acquisition of *omp*P2 gene obtained from Hi07 strain not only provided a resistance to chloramphenicol, but also increased the adhesion taxes especially in Hec1B cells.

*H. influenzae* type b P2 porine (341 amino acids, 37.782 Da) allows the diffusion of small molecules with a molecular weight of until 14000 Da to the periplasmatic space [38]. This is probably the antibiotics way of access into the bacterial cell. Small changes on the amino acid configuration provide different molecule captures by closing the canal, therefore modifying the porine function [39]. The basic amino acid changes in the porine overture loop domains 1 and 3 could difficult the penetration of chloramphenicol to the inner side of *H. influenzae*, resulting into a resistance to this antibiotic and also into a better adhesion to cell cultures.

The adhesion of mutant *H influenzae* strains on human cells surface is directly linked to the influence of graphene oxide nanoparticles on bacterial transformation, once the resulting mutants presented a higher adhesion to cell lines compared to wild type strains. Some studies have already related atmospheric pollution to cases of bacterial meningitis [40,41]. Therefore, this study corroborates to the hypothesis that links greater transformation frequency of naturally competent bacteria to the influence of nanoparticles and its consequences to the health of people exposed to them, especially in polluted and arid regions.

## Conclusions

Graphene oxide nanoparticles aid the transformation process, helping to increase the number of CFUs, and the mutants generated with the *omp*P2 gene from a *H. influenzae* resistant strain not only present an chloramphenicol resistance but also have an increased adherence patterns in A549 and Hec1B cell lines. Also, the increase of transformation frequency have some relationships with the adhesion, or the virulence of this bacterium. Then a gene liked to antibiotic resistance acquisition showed a great capacity of not only antibiotic transference characteristics between *H. influezae* strains as the transference of the virulence factors present in different strains in human upper respiratory tract.

## Competing interests

All the authors declare that they have no competing interests in this work.

## Authors’ contributions

JNV carried out the Molecular Biology design and plasmids; MCKA carried out the results analysis and revised the manuscript; RFCP carried out the cellular assays tests; LMH carried out the molecular genetics studies; HJC carried out the graphene synthesis and FESEM, HRTEM and Raman tests; VB participated in the drafting of the manuscript and gave technical support in Nanoengineering; ML carried out the molecular genetics studies and also the draft of the manuscript. All the authors read and approved the final manuscript.
